# Neuropsychological Deficits in Mice Depleted of the Schizophrenia Susceptibility Gene *CSMD1*

**DOI:** 10.1371/journal.pone.0079501

**Published:** 2013-11-14

**Authors:** Vidar M. Steen, Chirag Nepal, Kari M. Ersland, Rita Holdhus, Marianne Nævdal, Siri M. Ratvik, Silje Skrede, Bjarte Håvik

**Affiliations:** 1 Dr E. Martens Research Group for Biological Psychiatry and K.G. Jebsen Centre for Psychosis Research, Department of Clinical Science, University of Bergen, Bergen, Norway; 2 Center for Medical Genetics and Molecular Medicine, Haukeland University Hospital, Bergen, Norway; University of Wuerzburg, Germany

## Abstract

Recent meta-analyses of schizophrenia genome-wide association studies (GWASs) have identified the *CUB and SUSHI multiple domains 1* (*CSMD1*) gene as a statistically strong risk factor. CSMD1 is a complement control-related protein suggested to inhibit the classical complement pathway, being expressed in developing neurons. However, expression of *CSMD1* is largely uncharacterized and relevance for behavioral phenotypes is not previously demonstrated. Here, we assess neuropsychological behaviors of a *Csmd1* knockout (KO) mouse in a selection of standard behavioral tests. Deregulation of neuropsychological responses were observed in both the open field and the elevated plus maze tests, in which KO mice spent 55% and 33% less time than WT littermate mice in open areas, respectively. Altered behaviors were also observed in tail suspension and to higher acoustic stimuli, for which *Csmd1* KO mice showed helplessness and moderate increase in startle amplitude, respectively. Furthermore, *Csmd1* KO mice also displayed increased weight-gain and glucose tolerance, similar to a major phenotype of the metabolic syndrome that also has been associated to the human *CSMD1* locus. Consistent with a role in the control of behaviors, *Csmd1* was found highly expressed in the central nervous system (CNS), and with some expression in visceral fat and ovary, under tissue-specific control by a novel promoter-associated lncRNA. In summary, disruption of *Csmd1* induces behaviors reminiscent of blunted emotional responses, anxiety and depression. These observations suggest an influence of the *CSMD1* schizophrenia susceptibility gene on psychopathology and endophenotypes of the negative symptom spectra.

## Introduction

Schizophrenia is a severe psychiatric disorder of high heritability with a lifetime risk of approximately 1%. Onset of symptoms typically occurs in young adulthood, with chronic deregulation of behavior and emotions, including psychotic symptoms (hallucinations and delusions), emotional flattening and cognitive dysfunctions. Affective symptoms and anxiety are also regarded as key findings in this complex disorder [1]. The etiology of schizophrenia is thought to have a neurodevelopmental basis that disrupts neuronal plasticity and transmission, but the identification of disease mechanism has proven difficult, not least due to the phenotypic heterogeneity and polygenetic contributions. Still, alterations in immunity-related processes in schizophrenia have been documented in both epidemiological and genetic studies conducted over the past decades [2], [3]. While it is clear that complex genetic and environmental factors contribute to disease risk [4], the effect of specific immune-related genes on neuropsychological behaviors remains to be elucidated.

We previously found that the activity of complement-related factors (including complement component C3) was induced during long-term potentiation (LTP) of synaptic strength in the hippocampus of awake rats – a mechanistic model of learning and memory [5]. Since dysfunction in synaptic activity and cognitive abilities has been recognized as a primary and enduring core deficit in schizophrenia, we examined a set of complement control-related genes as potential susceptibility factors in schizophrenia, and identified statistically strong genetic associations to the homologous genes *CSMD1* and *CSMD2*, as well as to the *CR1* locus (C3 receptors) [6]. In parallel, the Psychiatric Genome-Wide Association Study (GWAS) Consortium (*PGC*) identified *CSMD1* as one of only seven verified loci in a large schizophrenia GWAS-based meta-analysis involving about 18.000 patients and 34.000 controls [7].

The *CSMD1* gene encodes a complement control-related protein with multiple copies of CUB and Sushi, a single transmembrane domain and an intracellular signaling peptide. The function of the CSMD1 protein is sparsely described, but inhibition of complement C3 activation is demonstrated *in vitro* [8], [9]. Although a possible relevance of *CSMD1* has been reported in autoimmune disease (neonatal lupus) [10], the precise role of *CSMD1* in immune responses remains to be further described. Distinct from a role in classic immunological pathways, it has also become clear that complement activity is tightly controlled in the brain, regulating C3/CR3-dependent axonal pruning by phagocytic microglia. This mechanism ensures precise wiring of neuronal circuits in the visual system during development [11]. It is therefore believed that deregulation of complement could lead to aberrant synaptic elimination also in other parts of the brain, which may influence the risk of both neurodegenerative and psychiatric disorders [12].

Further suggesting a role of *CSMD1* in psychopathology, GWAS studies on bipolar disorder (BIP) and major depressive disorder (MDD) also include nominal significant associations of genetic markers in *CSMD1,* which can be found in supplemental data in relevant literature [13]–[15]. BIP, MDD and schizophrenia are presently regarded as related disorders [4], [16] with partial overlap of symptom spectra including emotional flattening, depressive features and anxiety. It is postulated that the etiology of related psychiatric disorders depends on heritable endophenotypes that partially penetrate across disease borders, as well as on genetic risk factors that are selective to a particular disease [17]. We have suggested that the complement control-related *CSMD1* may affect shared symptomology in several psychiatric disorders [6]. However, the specific effect of complement factors on neuropsychological behavior is not known, and is very difficult to dissect in humans. We therefore sought to investigate the relevance of the *CSMD1* expression on neuropsychological endophenotypes using gene knock out (KO) mice. In the current study, we describe a constitutive *Csmd1* KO mice and determine the effect of *Csmd1* expression on behaviors using a selection of classical neuropsychological tests.

## Results

### Constitutive Disruption of *Csmd1* Expression in Mice

**Figure 1:**
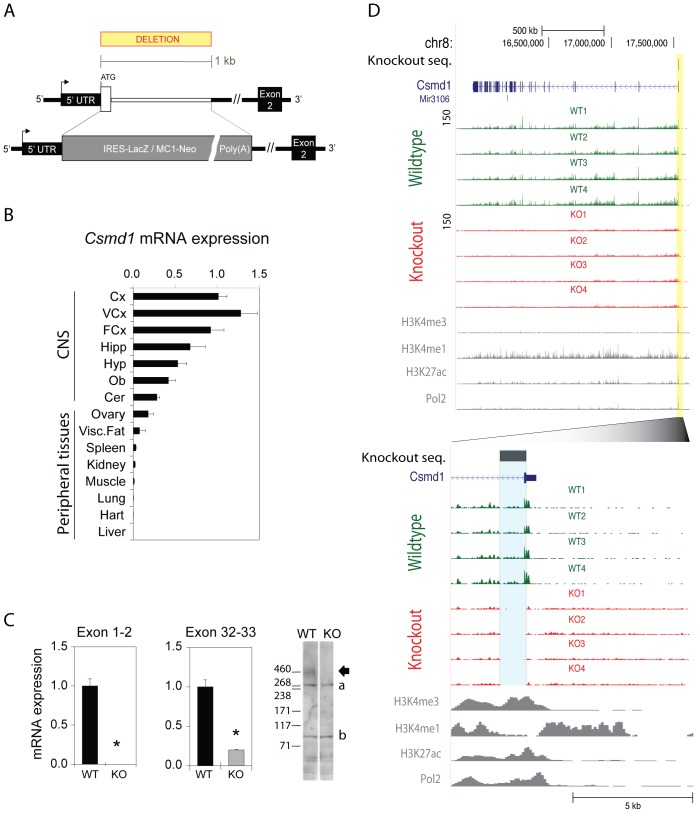
*Csmd1* RNA and protein expression in *Csmd1* knock-out and wild-type mice. (A) Schematic representation of the KO-strategy. A 1 kb genomic region (white lines) of exon1/intron1 was replaced with a selection cassette (grey box). (B) Expression of *Csmd1* mRNA measured by QPCR in an adult mouse tissue panel. *Csmd1* is predominantly expressed in brain tissues as compared to peripheral tissues. The highest expression level was identified in areas of the cortex. (C) Depletion of *Csmd1* mRNA in the cortex was documented by two exon-exon specific QPCR assays. Transcription of exon 1–2 was depleted, while about 20% residual expression could be observed when amplifying exon 32–33. KO mice lacked a protein band of expected size (389 KDa, arrow), as demonstrated by immunoblotting. Signals of lower molecular weight are indicated (a and b). (D) Mapping of RNA-seq reads to the *Csmd1* locus. RNA sequencing of cortex is shown for 4wild-type (green) and 4 *Csmd1* KO (red) mice (transcript scale: 0–150 reads). Coverage signals of modified nucleosomes (H3K4me3, H3K4me1 and H3K27Ac) and polymerase-2 binding profiles are shown for the mouse cortex. The 1 kb deleted sequence of *Csmd1* is highlighted in yellow (upper panel) and blue (lower panel). No RNA reads were mapped to the deleted genomic region in the KO mice. Abbreviations: Cx, cortex; VCx, visual cortex; FCx, frontal cortex; Hipp, hippocampus; Hyp, hypothalamus; Ob, olfactory bulb; Cer, cerebellum; Visc. Fat, visceral fat.

To investigate the functional relevance of *Csmd1* in the regulation of neuropsychological behaviors, we used a constitutive gene deletion to disrupt *Csmd1* gene expression in mice. The Reference Sequence collection describes mouse *Csmd1* as a 1.65 Mb long and 72 exon-rich gene, which encodes a 14 kb mRNA. To block expression through a minimal transgenic manipulation, a constitutive knockout (KO) of a 1 kb sequence from *Csmd1* exon1/intron1 was generated (Figure 1A). In WT mice, we found *Csmd1* mRNA to be expressed in the central nervous system (CNS) with highest levels in the cortex, while expression in peripheral tissues was not observed except for low levels in visceral fat and ovary (Figure 1B). In the KO mice, depletion of *Csmd1* mRNA and protein expression was documented by QPCR (exon1–2 junction-specific assay) and immunoblotting (custom-made goat anti-Csmd1 antibody) on cortex samples, respectively (Figure 1C). Consistent with disrupted *Csmd1* expression (Figure 1C), the deleted genomic DNA region was shown to be the major site for Pol2 binding, and H3K4me1, H3K4me3 and H2K27Ac modified nucleosomes – physical features of core-promoter activity (Figure 1D) [18]. Exact and complete removal of the targeted genomic DNA sequence was confirmed by specific loss of RNA products complementary to the deleted DNA region, as shown by comparing RNA sequencing data of individual *Csmd1* KO (n = 4) and WT (n = 4) mice to the mouse genomic reference sequence, respectively (Figure 1D).

**Figure 2:**
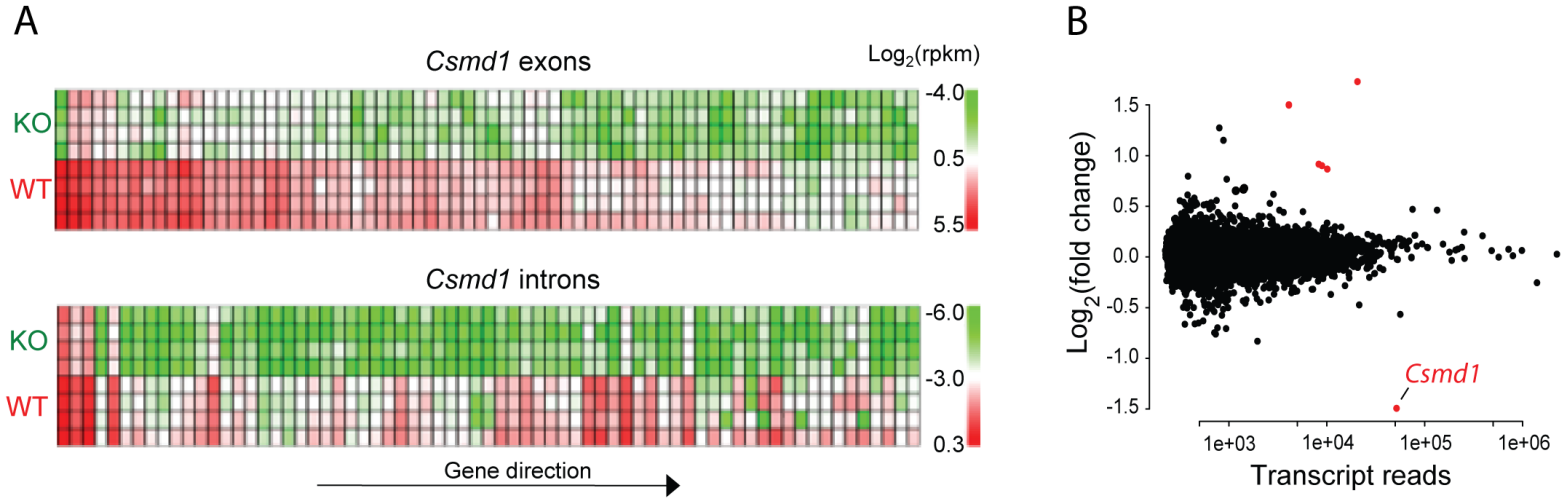
Transcriptome analyses by RNA sequencing of cortex from *Csmd1* KO and WT mice. (A) Heat map of RNA-seq expression levels, as measured by rpkm, mapping to individual exons and introns of *Csmd1*. The arrow indicates the orientation of exons and introns in their increasing number. (B) Analysis of differentially expressed RNAs. Significantly up-regulated and down-regulated transcripts are represented by red dots. Y-axis indicates the log2 (fold change).

The *Csmd1* locus consists of 72 exons and several long intronic regions. In *Csmd1* KO mice, some remaining transcription could be identified further downstream of the transcriptional start site (TSS) in exon 1, as documented by QPCR (exon 32–33 specific assay) (Figure 1C). We therefore examined how the exon1/intron1 deletion affected the transcription across the entire locus by analyzing the RNA sequencing data. We identified markedly reduced RNA transcription constitutive to all exons and introns of *Csmd1* (Figure 2A). Both QPCR and RNA sequencing suggest at least 60–70% reduction in the expression of *Csmd1* in the 3′ end of the gene, but we have not linked remaining expression to alternative *Csmd1* transcripts.

**Figure 3:**
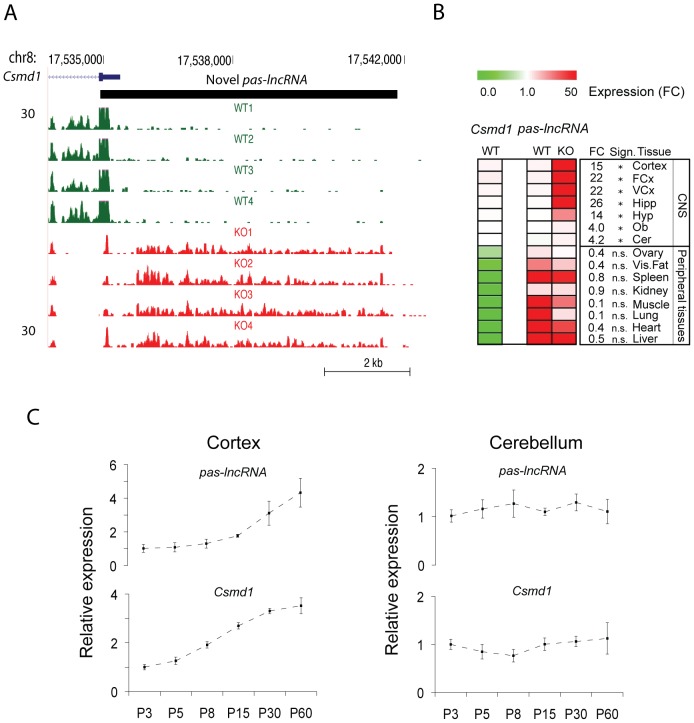
Expression of *Csmd1* promoter-associated long non-coding RNA (*pas-lncRNA*) in mouse tissues. (A) Map of RNA sequencing reads aligned to the promoter region of *Csmd1.* Data from individual *Csmd1* KO (N = 4; red lines) and WT (N = 4; green lines) mice are shown. The novel *pas-lncRNA* RNA (black thick line) is expressed antisense to the *Csmd1* promoter sequence (transcript scale: 0–30 reads). (B) Heat map representing the relative expression level of *pas-lncRNA* and *Csmd1* mRNA in peripheral and CNS tissues of *Csmd1* KO and WT mice, respectively. Expression values of each transcript are calculated relative to their respective expression level in cortex of WT mice. *pas-lncRNA* expression was induced in the CNS but not in peripheral tissues of *Csmd1* KO mice. Fold change values (FC) and statistical significances are listed for each tissue. (C) Co-regulated expression of *Csmd1* and pas-lncRNA in the developing cortex and cerebellum (*Csmd1*:*pas-lncRNA* expression correlation coefficient, cortex: r^2^ = 0.92). Y-axis represents relative expression level of RNA. X-axis indicates the postnatal day. Abbreviations: asterisk, t-test *P*-value<0.05; n.s.; not significant.

To clarify that *Csmd1* is the major deregulated genomic component of *Csmd1* KO mice, we analyzed the RNA transcriptome sequences for differentially expressed genes: *Csmd1* was the only significantly down-regulated gene in KO mice (Figure 2B). Overall, we observed a remarkably stable transcriptome profile, but noticed up-regulation of long non-coding RNAs (lncRNA) from two separate loci on chromosome 4 and 8, respectively (expression fold change KO/WT, range = 1.8–3.3; *P*-values<0.05) in the KO mice (Figure 2B, Table S1 in file S1). The identified lncRNAs are not functionally annotated in the literature. We also constructed and explored the expression profile of novel transcripts, finding only one *Csmd1* promoter-associated (pas) long non-coding (lnc) RNA to be differentially expressed (expression fold change KO/WT = 15±2.3; *P*-value<0.05) (Figure 3A). The pas-lncRNA is expressed antisense to the *Csmd1* promoter sequence and display co-regulated expression with *Csmd1* specifically in the adult and developing CNS (Figure 3B and 3C). In contrast, in peripheral tissues (where *Csmd1* mRNA expression levels are low) the expression of pas-lncRNA was constitutively high (Figure 3B). Thus, the expression of pas-lncRNA may reflect a mechanism for tight control of *Csmd1* promoter-activity specifically in the CNS.

### Behavioral Characterization of *Csmd1* Knockout Mice

A preliminary screen of health status and fertility identified no major defects in the *Csmd1* KO mice (see Materials and methods). However, preliminary examinations of the original repository *Csmd1* KO model revealed possible neuropsychological dysfunctions (n = 4–8 mice/genotype) (Figure S1 in file S1). Although with borderline statistical significance and low numbers (n = 4–8), the KO genotype was associated with marked effects in the open field test (50% reduced time in the open center, *P-*value = 0.1; Figure S1A in file S1), the tail suspension test (56% increased immobility time, *P*-value = 0.1; Figure S1B in file S1) and in the response to acoustic stimuli (59% increased startle amplitude, *P*-value = 0.1; Figure S1C in file S1).

We therefore performed a comprehensive analysis of behaviors and assessed a larger number of homozygous *Csmd1* KO and WT littermate mice, using a separate line of mice on a similar mixed genetic background (n = 23–24 mice/genotype). Mice were generated in one breeding scheme using heterozygote female and heterozygote male mice (except for the EPM test). Testing of littermate offspring started at age 10 weeks. Non-stressful tests were conducted on the same set of animals with a one-week interval in the sequence considered most optimal (detailed in Materials and methods). We also recorded diurnal locomotor activity and metabolism for each animal, to control for underlying phenotypes that could interfere with the behavioral assessment in specific tests.

### *Csmd1* KO Mice Avoid Open Space in the Open Field and Elevated Plus Maze Tests

**Figure 4:**
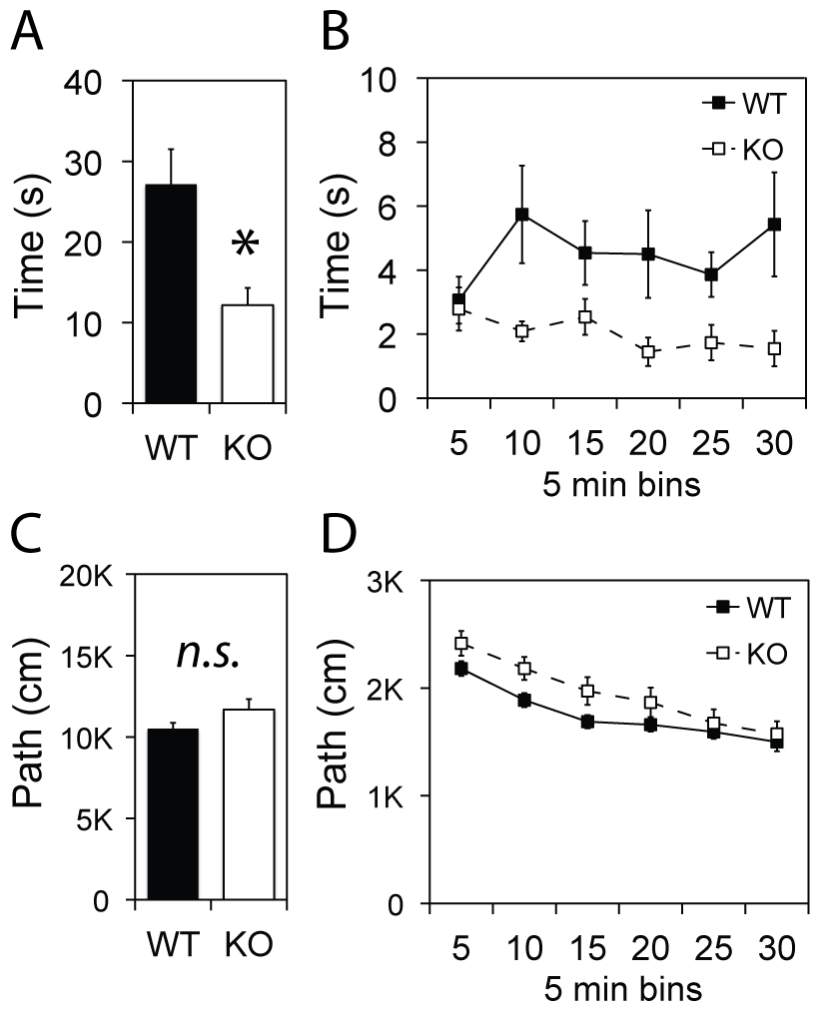
Behavior of *Csmd1* KO and WT mice in the open field arena. (A) Total time spent in the center of the arena is shown for KO and WT mice, respectively. (B) Sequential time bin analysis demonstrates that WT mice adapt to the test arena after the first time bin, while KO mice avoid the center throughout the test period. (C and D) Comparison of total path length and sequential bin analysis of path length demonstrate no statistically significant difference between KO and WT mice. Abbreviations: n.s., not significant; asterisk, statistically significant *P*-value<0.05; s, seconds.

The effect of *Csmd1* on the free adaption and exploration of a novel environment was first investigated by introducing the mice to the open filed (OF) arena: The time spent in the center of the field (open/exposed area) as opposed to a path along the borders (close to walls and corners) was recorded. *Csmd1* KO mice spent 55% less time in the center (average time ± s.e.m: WT: 27±4.3 sec., KO: 12±2.1 sec.; genotype group *P*-value = 0.003), but travelled a similar total distance during the test period, as compared to the WT mice (Figure 4A and 4C). *Csmd1* KO and WT mice also displayed a marked difference in adaption to the test arena following the initial exposure (first time bin) (Figure 4B and D): WT mice habituated to the OF center during the first period, and thereafter spent up to 80% more time exploring the center area. In contrast, *Csmd1* KO mice gradually avoided the center and ended the test by spending 43% less time in the center (last vs. first time bin).

**Figure 5:**
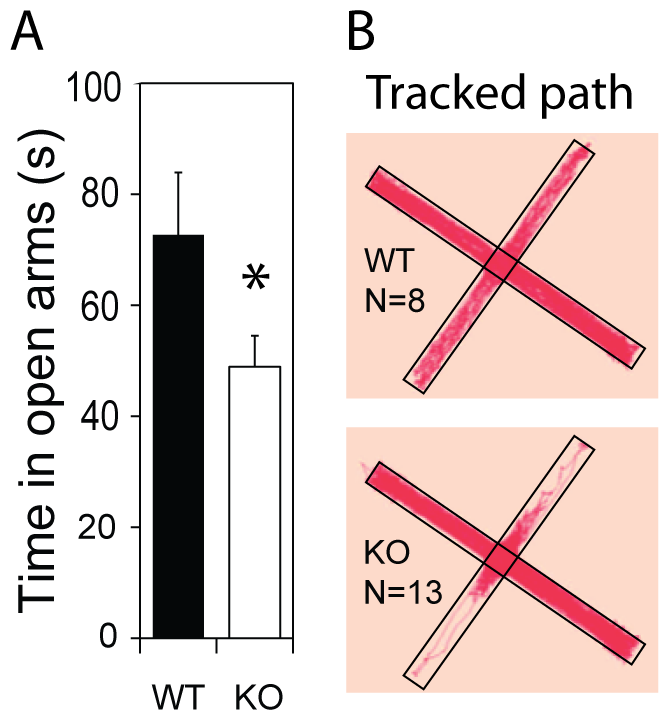
Behavior of *Csmd1* KO and WT mice in the elevated plus maze. (A) Analysis of total time spent on open arms demonstrate significant less time for KO mice (N = 13) as compared to WT mice (N = 8). (B) Compiled tracks from all mice show that *Csmd1* KO mice avoid entering open arms, as opposed to WT mice traveling over the entire test arena. Abbreviations: asterisk, statistically significant (*P*-value<0.05); s, seconds.

To replicate the effect of *Csmd1* expression on the exploration of new and open arenas, we examined the behavior of a separate (but smaller) set of *Csmd1* KO and WT littermate control mice in the elevated plus maze (EPM). In the EPM, mice are free to enter “closed” arms (corridors with high walls) or “open” arms (exposed platforms). While WT mice explored both corridors and platforms, *Csmd1* KO mice spent 33% less time on the open platforms (average time on platform ± s.e.m = WT: 73±11, KO: 48±5.3; genotype group *P*-value = 0.001) (Figure 5A) and stopped exploration (froze) just after crossing the entry point (Figure 5B). The marked avoidance of exploring the open platforms occurred despite *Csmd1* KO and WT mice showing similar numbers of open platform entries (average “open” platform entries ± s.e.m = WT: 15±2.0, KO: 13±1.7; genotype group *P*-value = n.s.).

### Deregulated Behaviors in *Csmd1* KO Mice in Response to Acoustic Startle Stimuli and Tail Suspension Tests

**Figure 6:**
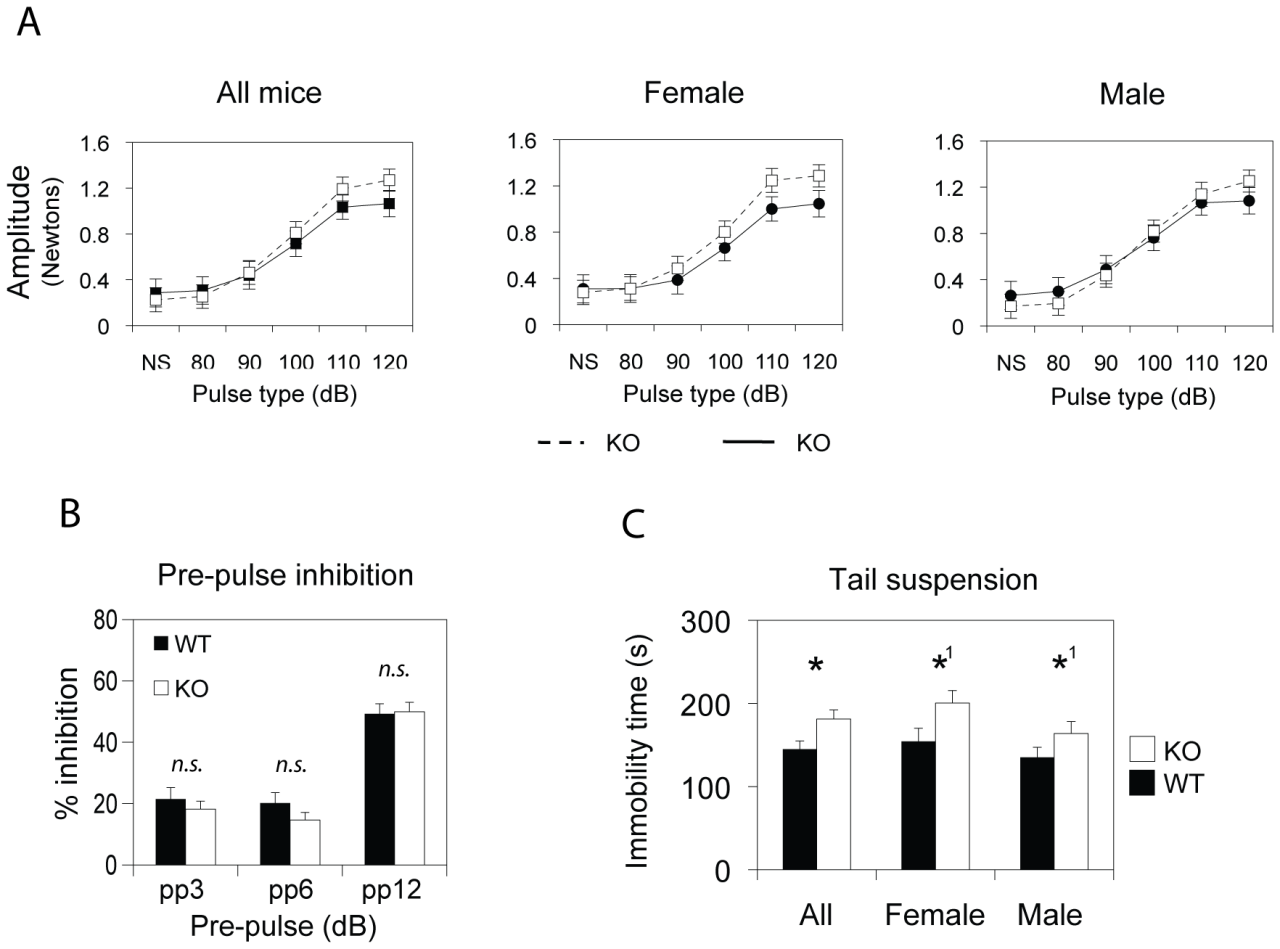
Behavior of *Csmd1* KO and WT mice to acoustic stimuli and tail suspension. (A) Startle responses of *Csmd1* KO mice in response to acoustic stimuli in the range of 80–120 dB, as compared to WT mice. The startle baseline was similar in male and female WT mice. A marginal increase in startle responses could be observed for higher acoustic stimuli in both genders of *Csmd1* KO mice, reaching statistical significance when analyzing all mice together (genotype-group interaction *P*-value<0.05). (B) There was no difference in amplitude response between KO and WT mice in the degree of inhibition after pre-pulse inhibition. (C) Tail suspension test demonstrated longer accumulated time immobile for KO mice as compared WT mice (P-value<0.05). Borderline statistical significance was observed for male *Csmd1* KO mice as compared to WT mice (P-value = 0.1).

Assessment of the acoustic startle reflex in mice has also been used to measure anxiety-like behaviors. Pre-pulse inhibition (PPI) of acoustic startle response may further indicate defects in the gating of neuronal signals and is often identified in candidate genes of psychotic disorders [19]–[21]. We therefore measured basal startle responses in *Csmd1* KO and WT mice, finding a moderate, but significant elevation of startle amplitude to higher acoustic stimuli in *Csmd1* KO mice (both genders combined; *P*-value = 0.043) (Figure 6A). Examination of PPI of the startling amplitude in *Csmd1* depleted mice showed no impairment, as normalized to the basal startle reflex (Figure 6B).

The effect of *Csmd1* on affect-like response was analyzed by the tail suspension test (TST), measuring the total time spent immobile. The test is classically used to investigate the effect of anti-depressant drugs and associates immobility with features of depression (learned helplessness) [22]. In suspended position, the *Csmd1* KO mice spent 25% more time immobile as compared to WT littermates (both genders combined) (average time immobile ± s.e.m = WT: 144±11.3, KO: 181±11.2; genotype group *P*-value = 0.02) (Figure 6C). This finding may link *Csmd1* expression to the state of learned helplessness.

### *Csmd1* KO Mice Display Increased Exploratory Activity but show Retained Memory in the Novel Object Recognition Test

**Figure 7:**
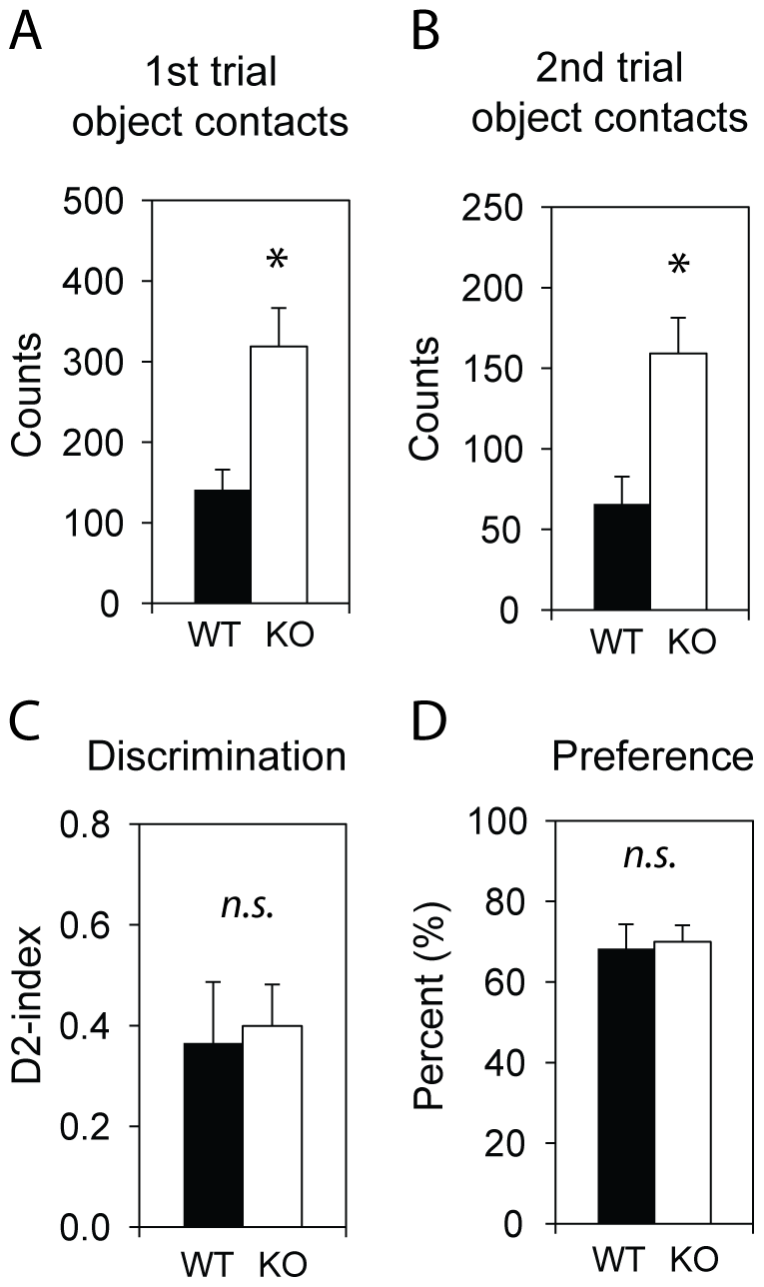
Analysis of object recognition memory in Csmd1 KO and WT mice. (A and B) Object contacts in the first and second trial of exposure to novel and familiar objects demonstrated increased contact counts for *Csmd1* KO mice (P-value<0.05). (B and C) Discrimination and preference analysis demonstrated no effect of *Csmd1* on object recognition. Abbreviation: n.s., not significant; asterisk, statistically significant (*P*-value<0.05).

The effect of *Csmd1* was also assessed for the cognitive process of recognition memory, as we previously identified a possible role of complement control-related genes in LTP [5]. The preference and discrimination between novel and familiar objects was measured. Notably, the *Csmd1* KO mice displayed a marked increase in exploratory activity to both familiar and novel objects, as compared to WT mice (2.4 fold increase, *P*-values<0.0001 in both 1^st^ and 2^nd^ trail; Figure 7A and B). The behavior of *Csmd1* KO mice reflects frequent nose contacts, and not a mere effect of the time spent at each object. With respect to object recognition, the test displayed no difference in working memory, illustrated by similar preference and discrimination (D^2^) indexes between mice genotypes (Figure 7C and 7D). However, we cannot rule out interference as mediated by e.g. increased exploration or anxiety- and depression-like behaviors (as documented in Figures 4 and 6).

### Increased Body Weight in *Csmd1* KO Mice

After completing the assessment of behaviors in OF, NORT, PPI and TST, we examined the behavior of *Csmd1* KO and WT mice outside the test arenas. The diurnal pattern of ambulatory, non-ambulatory (grooming), rearing, licking (drinking) and metabolic activities were measured in a Comprehensive Cage Monitoring System (CCMS) using non-invasive automated recordings.

The CCMS-based analyses revealed some genotype-specific activities in the dark-period only, but not of equal effect in both genders (Figure S2 in file S1): In the dark period, *Csmd1* depletion increased the total horizontal activity counts in male mice (Figure S2A in file S1). The increase in activity was specific for ambulatory movements (68–76% increase, male-genotype group *P*-value = 0.004). In contrast, the total horizontal activity of female *Csmd1* KO mice remained unaffected (Figure S2B in file S1), but with a small reduction in non-ambulatory movements in the dark periods only (28–52% decrease, female-genotype group *P*-value = 0.03).

Changes in diurnal activity were gender and dark period specific, and therefore not believed to interfere with neuropsychological behaviors observed in the standard tests. The standard tests were performed during defined times of the light periods and the effect of *Csmd1* depletion on neuropsychological behaviors was identified in both genders.

We routinely monitored the weight of all animals during regular handling and when examining behaviors (water and food *ad libitum*). We identified a significant accumulation of body weight over time, especially in female mice (% average weight gain ± s.e.m after 18 weeks: male mice: 12.8±0.12, genotype group *P*-value = 0.01; female mice: 21.2±0.14, genotype group *P*-value = 0.01) (Figure S3A in file S1). To gain further insight into the metabolic phenotype, we measured the effect of *Csmd1* expression on glucose tolerance, finding increased glucose tolerance in *Csmd1* KO mice as compared to WT mice (Figure S3B in file S1). CCMS-based measurements of metabolic rate (CO_2_ production) did not differ between *Csmd1* KO and WT mice (data not shown).

## Discussion

In this study, we provide an important biological validation of the *CSMD1* schizophrenia susceptibility gene by demonstrating that *Csmd1* expression affects the deregulation of neuropsychological behaviors in mice, thereby showing a potential functional relevance of the this gene locus in psychopathology. Using a transgenic mouse line depleted of *Csmd1* expression, we demonstrate that *Csmd1* is primarily expressed in the brain (Figure 1), and that loss of expression induces agoraphobic-like responses in the open field and elevated plus maze apparatuses, as well as a blunted response (learned helplessness) upon tail suspension (Figures 4, 5 and 6). These tests are classically used to measure the effect of substances developed for the treatment of anxiety and depression [22], 23. Although it may be difficult to pinpoint the precise human phenotypic counterparts, the behavioral assessment of *Csmd1* KO mice could be relevant for endophenotypes of complex psychiatric conditions, including parts of the negative and affective responses of schizophrenia which significantly contribute to impaired quality of life in these patients [1], [24].

The observation of anxiety- and depression-like behaviors in *Csmd1* KO mice may also provide one example supporting the notion of shared genetic susceptibility across schizophrenia, bipolar disorders and major depression [16]. These are heritable and related disorders with partial overlap of symptom spectra including emotional flattening, depressive features and anxiety. Genetic associations to *CSMD1* can be found in all three disorders (as found among association data reported in supplemental information of [13]–[15]).

The relevance of *Csmd1* for brain functions was further emphasized by the identification of a *Csmd1* promoter-associated lncRNA, possibly responsible for brain-specific promoter activity in the adult and developing CNS (Figure 3). Such promoter-associated lncRNAs is a new class of long untranslated RNA which may underlie a functional coupling of transcription and translation/splicing [25], [26]. Finally, we also demonstrate that depletion of *Csmd1* has a remarkably limited effect on the whole transcriptome (Figure 2). Together, these findings suggest a key role of *Csmd1* itself on the control of neuropsychological behaviors and provide a new framework for understanding how *CSMD1* misexpression may lead to onset of neuropsychiatric disorders.

When assessing the memory performance of KO mice, we observed no effect of *Csmd1* expression on object discrimination of preference. However, we did observe a large and highly significant increased exploration of objects (both familiar and new) in *Csmd1* KO mice (Figure 7). Retained memory performance may seem unexpected, since we have previously linked the expression of complement control-related genes to LTP [5]. However, we cannot rule out that increased object contacts (exploration) are due to the observed agoraphobia-like behavior of *Csmd1* KO mice, which leads to avoidance of open space and, therefore, might mask possible defects in object preference in the KO mice.

Recently, a similar *Csmd1* KO introduced on a mixed B6∶129 (B6 strain unknown) mouse genetic background was assessed for changes with respect to behaviors of social interaction, anhedonia, pre-pulse inhibition (PPI) of startle reflex and locomotor response to *d*-amphetamine [27]. These behaviors are often related to complex schizophrenia-like behaviors, but the study did not find any behavioral changes in *Csmd1* KO mice. PPI is the only test overlapping with our report, and we both find no genotypic effects. However, considering the possible association of human *CSMD1* with depression and anxiety-linked disorders with suspected genetic overlap to schizophrenia (see introduction), we tested relevant behaviors in *Csmd1* KO mice in response to basic startle response, open field, elevated plus maze and tails suspension. Although we assess different behavioral test than Distler et al., positive findings may also be influences by additional factors: The mice used in the two studies are derived and maintained on separate mouse strains. While we used RNA sequencing to confirm genomic depletion and mechanisms of CNS-specific expression, residual expression and KO of the *Csmd1* DNA segment is not characterized by genomic analyses by Distler et al. Also, we use a higher number of mice and analyze possible gender-mediated difference in behaviors. Analyzing high number of mice may be essential to compensate for increased behavioral variance that may be linked to heterozygous breeding patterns on mixed genetic backgrounds.

Furthermore, anxiety- and depression-like behaviors, i.e. phenotypes we identified in *Csmd1* KO mice, may mask the effects of the *Csmd1* KO in complex context-dependent tests like e.g. social learning. Also, we control for diurnal activity pattern of each mouse in our study, and therefore applied single housed mice prior to testing. Housing conditions may trigger some of the underlying susceptibility for neuropsychological deficits in *Csmd1* KO mice. Social isolation in both young and adult mice has in recent reports been demonstrated to be a relevant stressor generating deficits in the function of neuronal circuits and behaviors [28], [29]. The ability of *Csmd1* KO mice to capture the influence of environment (stressors) x gene interactions is to be examined in future studies.

The *Csmd1* KO mouse captures several major clinical phenotypes that have been associated to the human *CSMD1* locus, both psychiatric disorders and somatic conditions. The presence of weight gain and altered glucose tolerance in *Csmd1* KO mice is consistent with the strong association of *CSMD1* to the body-mass-index and measures of the metabolic syndrome in the large Framingham Heart Study [30]. This observation provides an important cue to further examine the effect of *Csmd1* on metabolism as part of a complex biological system [31], [32]. Metabolic disturbances are of major relevance for schizophrenia-spectrum disorders, and some studies have identified severe metabolic deregulations in patients [33]–[35]. However, it is still not fully understood how central and peripheral signaling pathways in combination with antipsychotic treatment and genetic susceptibility provoke such clinical symptoms [36]. A detailed analysis of possible CNS and peripheral tissue mechanisms responsible for the metabolic phenotype of *Csmd1* KO mice is to be explored in future studies.

An unresolved question is which molecular and cellular mechanisms mediate the influence of *Csmd1* on psychiatry-relevant behaviors. A prominent molecular feature of CSMD1 is the extracellular repeated pattern of CUB and Sushi, domains primarily found in complement- and development-related proteins [37], [38]. Many of these proteins assist, or “complement”, the process of clearing pathogens for destruction by other cells, as part of the innate immune system. In the peripheral immune system, the complement cascade is activated through the classical, alternative, or lectin pathways, all converging on the activation of complement C3 leading to the formation of the membrane attack complex [39]. Recombinant CSMD1 is reported to block complement C3 activation and cell clearance [8].

It has become increasingly clear that immune molecules, in addition to their roles in classical immunological pathways, contribute to brain development and function [40], [41]. Complement components are localized to developing synapses where they play an essential role for synaptic refinement and precise neuronal connectivity, and mice deficient in the classical complement cascade (C1q, C3 KOs) exhibit similar defects in synaptic pruning in the developing brain [42], [43]. In the brain, complement factors (C3, CR3) mediate microglia-synapse interaction and regulate activity-dependent synaptic pruning in the postnatal retinogeniculate system [11], implicating a key role of complement in protecting synapses from aberrant elimination. In the adult brain, we have demonstrated expression of similar molecules (complement and HLA) during hippocampal LTP [5].

Our identification of predominant expression of *Csmd1* in adult brain may therefore indicate a relevance of the complement pathway in neuronal processes underlying the risk of schizophrenia, and may contribute to further insight into the suspected link between immunological factors and disease risk in psychiatric disorders.

## Materials and Methods

These experiments were approved by the Norwegian Committee for Animal Research in accordance with European Community Council Directives.

### *Csmd1* Knockout Mice

A DNA sequence-verified repository *Csmd1* gene knock-out (KO) mouse model (clone TF0137, Taconic, Denmark) was generated with embryonic stem cells derived from 129SvEvBrd mice, in which a 1,070 bp genomic sequences of the exon 1– intron 1 junction was replaced with a LacZ/Neo selection cassette [44]. The selection cassette is expressed in frame with the starting protein-coding sequence of *Csmd1* in exon 1. In the present study, *Csmd1* knockout mice were bred on a mixed background of 129SvEvBrd:C57BL/6NTac and genotyped according to the provided protocol (Taconic, Denmark). We used a high number of mice in the breeding strategy and tested a high number of littermate offspring, to control for potential effects mediated by variance in the random genetic contribution form the two mouse strains. For this study, *Csmd1* KO and WT control mice were generated from the F2 generation using 30 heterozygote (HET) female mice and 15 HET male mice. From this we obtained 130 male (27% KO; 48% HET; 25% WT) and 141 female (20% KO; 50% HET; 30% WT) mice. Breeding of the *Csmd1* KO genotype followed Mendelian inheritance. For this initial characterization of *Csmd1* KO mice, we also controlled for genotypic effects on locomotor activity, which may interfere with the assessment of neuropsychological behaviors: Diurnal activities were measured in the compressive cage monitoring system (CCMS) for three days on single mice (See Supporting Information file S1). To enable comparisons between tests, we therefore used a testing paradigm in which mice were single housed 2 weeks before start of behavioral assessments.

Health status of the mouse line was examined by evaluating blood chemistry, cardiology, immunology, fertility, metabolism, oncology, ophthalmology and neurological behavior (n = 4–8 mice/genotype) (Taconic, Denmark). This preliminary study indicated that mice were in general healthy and fertile with 8–10 pups per litter, but showed signs of deregulation of specific neurological functions (See Supporting Information file S1).

### Tissue Collection, RNA Purification and QPCR

Mice were first sedated with Isoba®vet before being deeply anaesthetized by intraperitonal injection of Pentobarbital. Transcardial perfusion with ice-cold NaCl (9 mg/ml) was performed before dissection of tissues. Isolated tissue samples were homogenized with TissueLyser (QIAGEN) and RNA isolated by RNeasy Mini Kit (Sigma-Aldrich, Norway). The RNA concentration was measured using a NanoDrop ND-1000 spectrophotometer. cDNA was synthesized from 50 ng RNA with Superscript III First-Strand Synthesis System for RT–PCR (Life Technologies, Sweden), as previously described [5]. The relative gene expression levels were determined with the comparative Ct-method, and presented as fold change values normalized to *actb*. Endogenous control by actb was compared to *arbp* mRNA and eukaryotic 18S mRNA and found stable across multiple tissues (data not shown). The following PCR primers (forward, reverse) were used for quantitative SYBR green real-time RT–PCR (Eurogentec; purchased from Medprobe, Norway): *csmd1*∶5′-TGTGCGTGTGGAATATCTGC, 5′- AAGCCAGGACTTTCAATGG; *pas-lncRNA*: 5′-GCCACCATTGAAAGGAGGTA, 5′-CAGAAAGGCATAGCAAAGGC; *actb: 5′-* CTTCTCCAGGGAGGAAGAGG, 5′-TACAGCTTCACCACCACAGC.

### Immunoblotting

A custom made affinity-purified goat anti-Csmd1 antibody (21^st^ Century Biochemicals lnc., MA, USA) was generated against the peptide sequence: Nt-QRVTETLAAWNDHR (encoded by *Csmd1 exon 9*). The antibody detected a protein band corresponding to full-length Csmd1 protein, when using freshly prepared protein homogenates obtained from mouse cortical tissue. Tissue samples were homogenized in 500 µl RIPA (with Complete Mini, EDTA-free protease inhibitor) and protein concentrations were determined following the *DC* Protein Assay (Bio-Rad Laboratories AB, Norway). Proteins were separated on 3–8% Tris Acetate gel and transferred onto PVDF membranes (NuPage mini-gel system, Life Technologies, Sweden). Detection was performed with donkey anti-goat IgG–HRP (1∶10 000) and Enhanced Chemiluminescence (GE Healtcare, United Kingdom).

### RNA-sequencing and Bioinformatics Analyses

RNA was extracted from adult mouse cortex by RNeasy Mini Kit (Sigma-Aldrich, Norway). Ribosomal RNA was depleted with the RiboMinus™ Eukaryote Kit for RNA-Seq (Life Technologies, Sweden). Whole transcriptome cDNA was made from RNaseIII fragmented ribosomal depleted RNA following the protocol of the Library Builder™ Whole Transcriptome Core Kit (Life Technologies, Sweden). Samples were barcoded, pooled and sequenced on the SOLiD 5500xl system using a read length of 75 bp.

Adapters from the sequence reads were trimmed using custom perl scripts. We used TopHat 1.3.3 [45] to map the RNA-seq reads to the mouse (mm9 assembly) genome, with Refseq transcripts as reference gene model, by allowing a mismatch of 2 nt. Novel transcripts were constructed using the Cufflinks [46]. Differentially expressed genes were calculated at a false discovery rate of 1% using DESeq [47].

### Behavioral Testing

Neuropsychological behaviors were examined in a selection of standard tests using 23 *Csmd1* KO mice (11 males and 12 female) and 24 WT mice (12 males and 12 females). Mice were housed individually 2 weeks before start of the first test. Tests were performed in the following sequential order, starting at the age of 10 weeks: 1^st^ week: open field (OF); 2^nd^ week: novel object recognition test (NORT); 3^rd^ week: startle reflex and prepulse inhibition test (PPI); 4^th^ week: tail suspension test (TST); 5–6^th^ week: comprehensive cage monitoring system (CCMS). Testing was performed in the first phase of the light cycle between 6 AM and 12 PM, and standard acclimation procedures were applied for each behavioral test. The experiment was recorded and motion tracked by Viewpoint where applicable. Elevated plus maze test (EPM) was performed on a separate colony of female littermate mice, obtained from a heterozygous breeding strategy (8 *Csmd1* KO and 13 WT mice) and was tested for a period of 6 minutes in the EPM arena at 12 weeks of age. A brief description of behavioral tests can be found in Supporting Information file S1.

### Statistical Analysis

Statistical significance was evaluated with un-paired t-test or 2-way analysis of variance (ANOVA) as applicable. Significance threshold was set to *p*<0.05. Data are shown as mean ± SEM in figures or text. Behavioral data were analyzed with GraphPad Prism (San Diego, California, USA), and statistical significance for expression analyses was examined in Excel (Microsoft Corporation).

## Supporting Information

File S1 Supporting information.
(DOCX)
