# Supplementary material for: Neuropsychological Deficits in Mice Depleted of the Schizophrenia Susceptibility Gene CSMD1
Source: PLoS One. 2013 Nov 14;8(11):e79501. doi: 10.1371/journal.pone.0079501 (PMC3828352; doi:10.1371/journal.pone.0079501)
Supplement: File S1 — Supporting information. (DOCX) [file pone.0079501.s001.docx]

**SUPPORTING INFORMATION**

**Neuropsychological deficits in mice depleted of the schizophrenia susceptibility gene *CSMD1*.**

Vidar M. Steen^1,2^, Chirag Nepal^1,2^, Kari M. Ersland^1,2^, Rita Holdhus^1,2^, Marianne Nævdal^1,2^, Siri M. Ratvik^1,2^, Silje Skrede^1,2^, Bjarte Håvik^1,2^.

Addresses:

1. Dr E. Martens Research Group for Biological Psychiatry and K.G Jebsen Centre for Psychosis Research, Department of Clinical Science, University of Bergen, Norway.
2. Center for Medical Genetics and Molecular Medicine, Haukeland University Hospital, Bergen, Norway.

Correspondence: Dr. Bjarte Håvik, Department of Clinical Science, Haukeland

University Hospital, Laboratory building, N-5021 Bergen, Norway.

Tel: +47-55977033

E-mail: bjarte.haavik@uib.no

CONTENT:

**Supplemental Information – Table:**

Table S1: RNA sequencing page 2

**Supplemental Information – Figures:**

Figure S1: Behavioural tests page 3

Figure S2: CCMS page 4

Figure S3: Metabolism page 5

**Supplemental Information – Methods:**

Behavioral testing page 6

TABLE:

RNA sequencing

| **Chromosomal location of transcripts** | **Base Mean** | **Base Mean (WT)** | **Base Mean (KO)** | **log_2_FC** | **adjusted p-value** | **RNA** |
| --- | --- | --- | --- | --- | --- | --- |
|  |  |  |  |  |  |  |
| *Up-regulated genes* |  |  |  |  |  |  |
| chr4:41763294-42104673_NR_033506 | 20561 | 9526 | 31596 | 1.73 | 3.45E-10 | non-coding |
| chr4:42452733-42476567_NR_033123 | 4094 | 2136 | 6051 | 1.50 | 9.26E-11 |  |
| chr4:41902019-41925853_NR_033123 | 4095 | 2142 | 6048 | 1.50 | 9.12E-11 |  |
|  |  |  |  |  |  |  |
| chr8:19863864-19893010_NR_030708 | 8271 | 5731 | 10810 | 0.92 | 1.11E-18 | non-coding |
| chr8:19991156-20020278_NR_030708 | 8917 | 6220 | 11614 | 0.90 | 3.04E-18 |  |
| chr8:19981360-20020392_NR_028428 | 10114 | 7163 | 13064 | 0.87 | 6.93E-17 |  |
|  |  |  |  |  |  |  |
| *Down-regulated genes* |  |  |  |  |  |  |
| chr8:15892545-17535385_NM_053171 | 51526 | 76036 | 27015 | -1.49 | 2.31E-23 | Csmd1 |

**Table S1: List of differentially expressed genes in the cortex of *Csmd1* KO mice as compared to WT mice.** Gene expression levels were measured by RNA sequencing of cortical RNA isolated from *Csmd1* KO (n=4,) and WT (n=4) mice, using the SOLiD 5500xl system with 75bp read length. Mean expression levels across replicates (BaseMean) are listed in the table. Thresholds of log2 fold change >0.75, BaseMean expression > 1000 and adjusted *P*-value < 0.05 were applied. Up-regulated non-coding RNAs were encoded by two loci on chromosome 4 and 8, respectively. *Csmd1* was identified as the only down-regulated transcript.

FIGURES:


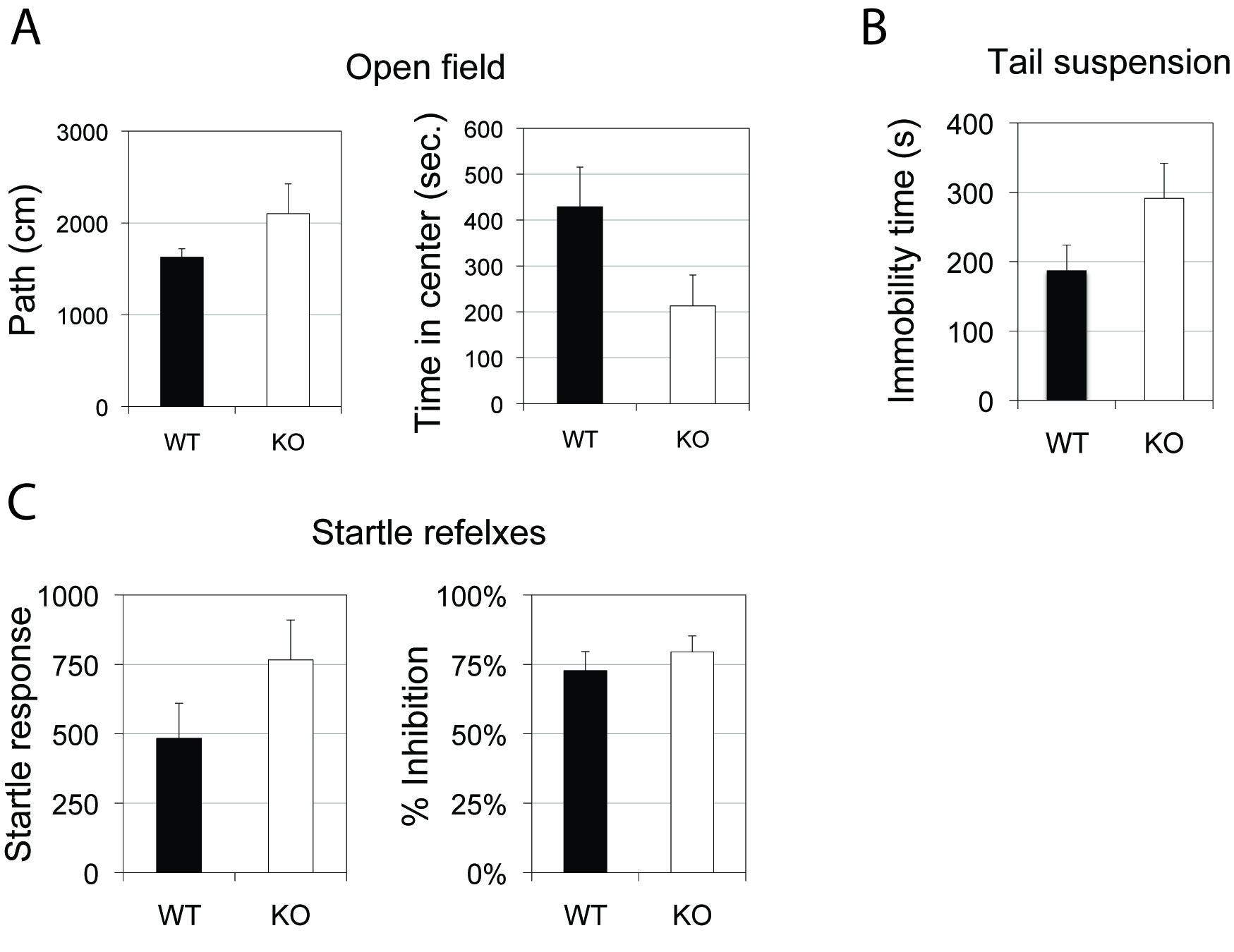


**Figure S1: Behavior of *Csmd1* KO and WT mice when exposed to the open field, tail suspension and acoustic stimuli.** The behavior of mice was examined in a preliminary test using 8 KO mice (4 male and 4 female) and 4 WT littermate control mice (2 male and 2 female). (A) In the open field test, KO mice travelled a similar distance (or slightly more) as WT control mice (P-value=0.3). KO mice spent 50% less time in the open centre as compared to WT control mice. Probably due to the inclusion of few mice, the effect was only borderline statistically significant (P-value=0.1). Similarly, KO mice also displayed 56 % increased immobility time in tail suspension (B; P-value=0.1) and 59% increased startle response to an acoustic stimuli pulse of 120 dB (C; P-value=0.1). No defects was observed in response to pre-pulse inhibition (C; P-value=0.4).

CCMS
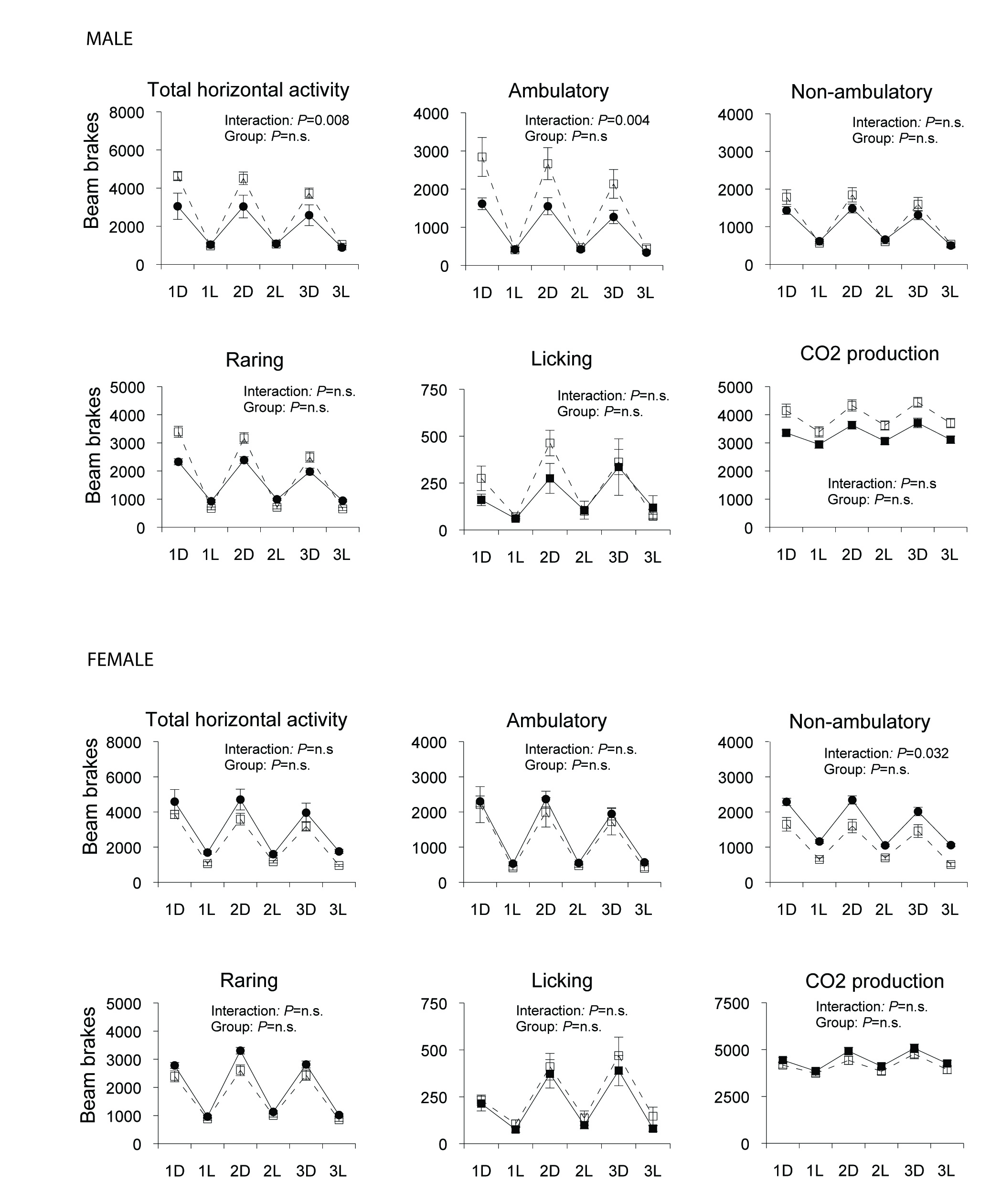


WT

KO

**Figure S2: Diurnal activities of *Csmd1* KO and WT mice measured in the Comprehensive Cage Monitoring System (CCMS) over a period of three days.** Raw-data is presented for results shown in Table 1. N = KO male: 12, KO female: 12, WT male 11, WT female: 12.

Metabolism


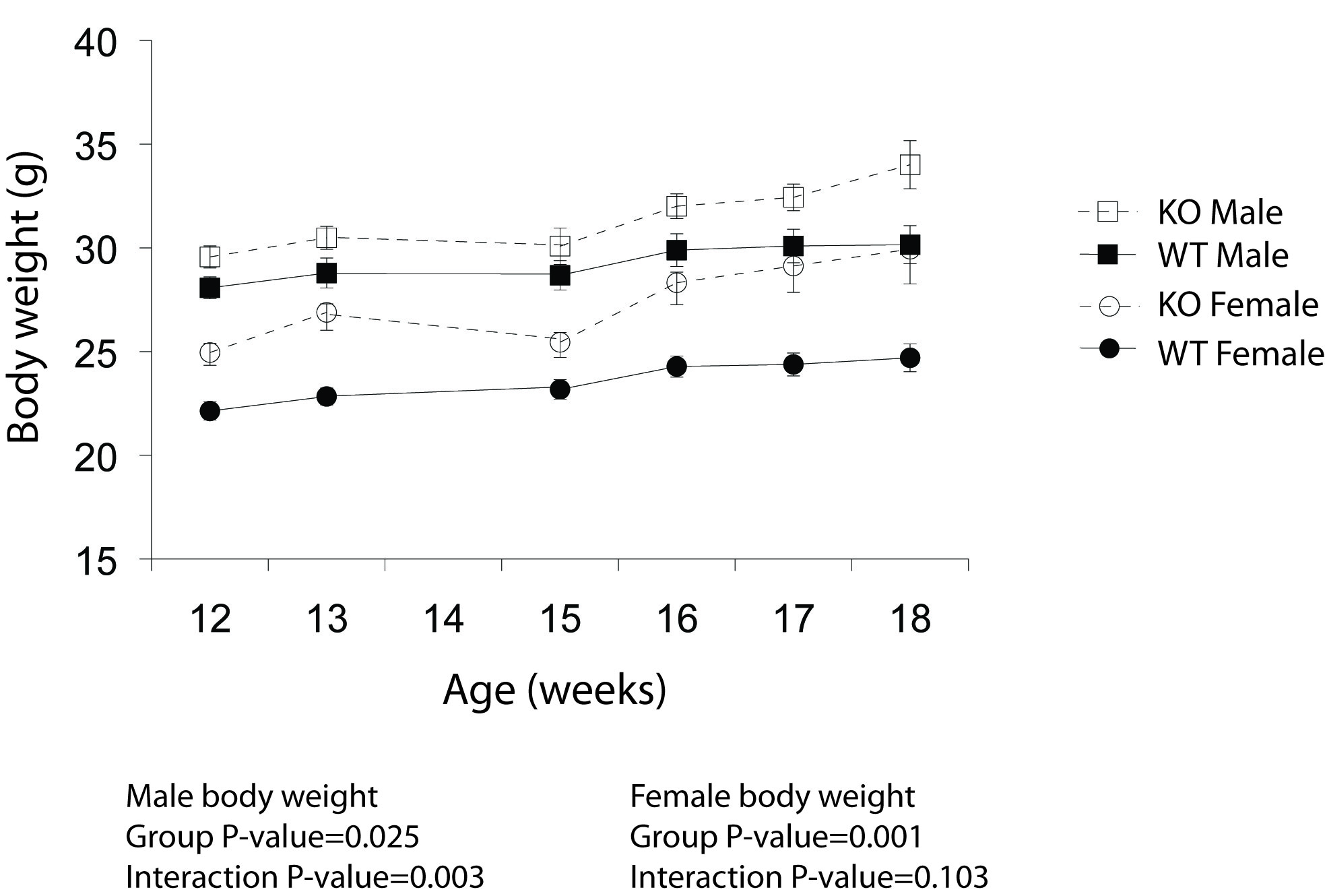
A

B


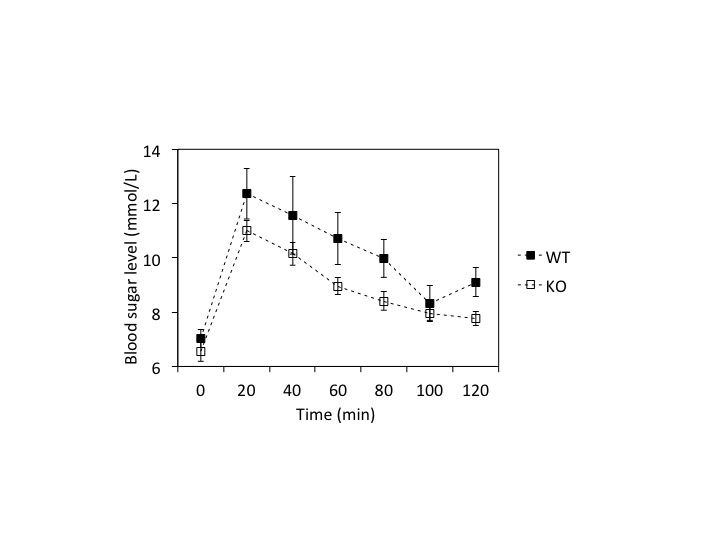


**Figure S3: Body weight and glucose tolerance of *Csmd1* KO and WT littermate mice.** (A) Body weight was measured during the course of behavioral testing. *Csmd1* KO mice had a statistically significant higher body weight than WT mice. The results are summarized in Table 1 and discussed in the text. N = KO male: 12, KO female: 12, WT male: 11, WT female: 12. (B) Time-course of blood glucose levels after glucose injection. The glucose level was lower in *Csmd1* KO mice as compared to WT littermates (group *P*-value = 0.06; interaction *P*-value<0.000). The glucose level in *Csmd1* KO and WT mice interacted after 100 minutes. N = WT: 8, KO: 13.

METHODS:

*Behavioral testing*

Neuropsychological behaviors were examined in a selection of standard tests. Briefly, the tests were conducted as follow:

Open Field (OF): The time and path length was recorded in the center (13.5 x 13.5 cm square) of the open field (50 x 50 cm square) during a test period of 30 minutes.

Novel Object Recognition Test (NORT): The nose of a mouse is tracked by Viewpoint and exploration time is recorded within 2 cm of the object. Pairs of objects were positioned in opposite quadrants of the open field 5 cm from each wall. Mice were placed in the open field with two similar objects for 20 minutes (trial 1). After a retention interval of 30 minuets mice were returned to the open field with one copy of the objects in trial 1 (familiar object, but using a third copy of the object in trial 1), and a novel object for 20 minutes (Trial 2). Familiar object: Corning cell culture flasks (75 cm2) filled with sand. Novel object: Clear glass mug. To evaluate non-spatial memory, the following measurements were recorded; e1: Total time spent exploring objects in trial 1; e2: Total time spent exploring objects in trial 2; D2 index: (time spent exploring novel objects – time spent exploring the familiar object)/2; Percent preference: (Time spent exploring novel object/Time spent exploring that familiar object) x 100.

Startle response and PPI: Mice were presented with 6 acoustic stimuli of different intensities (duration: 40 msec at 0, 80, 90, 110, and 120 dB). A total of 30 trials in a pseudo-randomized manner with an average inter-trial-interval (ITI) of 15 sec (range: 10-20 sec) and with ITI background noise of 70 dB. Amplitude of startle was measured within a 150 msec window following the stimuli. PPI was examined the following day using a prepulse of 20 msec with 3, 6 or 12 dB above background (70 dB) preceding the 40 msec long 120 dB pulse by 100 msec. 12 blocks of 10 pulse presentations (twice each of: no stimuli, 120dB alone or proceeded by the three types of prepulses) were recorded (first and last block with no prepulses).

Tail suspension test (TST): Mice were suspended by the tail using Scotch tape and immobility time was measured using an automated tail suspension test apparatus (Med-Associates) during a total test period of 6 minutes.

Comprehensive cage monitoring system (CCMS): Diurnal locomotor activity over three days was measured in the CCMS test apparatus (Columbus Instruments, Columbus, OH, USA) in a non-invasive and non-biased manner. Activity was recorded by infrared photocell sensors spaced at 1.27 cm, with 16 sensors in the x and z plane and 8 sensors in the y-axis. Automatic measurement of oxygen consumption by the CCMS failed due to technical reasons, but CO2 production (ml/kg/hr) was recorded.
